# Impact of Genes and Proportional Contribution of Parental Genotypes to Inheritance of Root Yield and Sugar Content in Diploid Hybrids of Sugar Beet

**DOI:** 10.1155/2014/580623

**Published:** 2014-01-28

**Authors:** Ivica Stancic, Jelica Zivic, Sasa Petrovic, Desimir Knezevic

**Affiliations:** ^1^College of Agriculture and Food Technology Prokuplje, Cirila and Metodija 1, 18400 Prokuplje, Serbia; ^2^Faculty of Agriculture, University of Pristina, Kosovska Mitrovica-Zubin Potok-Lesak-Kopaonicka bb, 38217 Lesak, Kosovo and Metohija, Serbia

## Abstract

This paper analyzes the impact of genes and proportional contribution of parental genotypes on the inheritance of root yield and sugar content in diploid hybrids of sugar beet. The survey included two diploid male-sterile monogerm lines and three single (SC) male-sterile hybrids as maternal components, while three multigerm diploids were used as pollinators. The partitioning of genotypic variance into additive and dominant components was performed by half sibling (HS) and full sibling (FS) covariance. The proportional contribution of individual components of crossbreeding (lines, testers, and interactions) was exhibited in the expression of certain characteristics of F_1_ generation. Genotypic variance components showed a significant effect of nonadditive gene action (dominance) in the inheritance of root yield and sugar content, while the additive effect of genes was less significant. Maternal components had a greater proportional contribution to root yield, while lines, pollinators, and their interactions had an equal contribution to sugar content.

## 1. Introduction

The genetic constitution of sugar beet varieties has changed from multigerm populations to monogerm anisoploid varieties and monogerm diploid and triploid hybrids developed due to the occurrence of monogerm, polyploidy, and male sterility.

In an analysis of the production values of diploid, triploid, and tetraploid hybrids [1], it was suggested that they can be highly productive, regardless of the type of hybridization. Furthermore, the author noted that the occurrence of heterosis was present in all types of hybridization, but it was more evident in diploid and triploid hybrids [2, 3]. When analyzing the results of research on creating monogerm diploid sugar beet hybrids [4], that monogerm diploid sugar beet hybrid hybrid Vitola characterized good combination of genes with impact to high root yield as well and other traits (good tolerance and partial resistance to beet necrotic yellow vein virus (BNYVV) and *Cercospora beticola* sacc.) and is suitable for cultivation in irrigation systems. Comparative analysis of diploid and triploid sugar beet hybrids was the subject of research of many authors [3, 5–7].

Sugar beet yield is a portion of root dry matter, with higher yields obtained with increasing amount of dry matter produced in the root [8]. Most plant breeders agree that genotype and the environment as well their interaction influence the expression of root yield [9]. Therefore, the highest root yield and quality of sugar were exhibited by some early genotypes in the harvest season [10], while some late harvest genotypes resulted in greater root yield and higher sugar content than earlier harvest genotypes [11]. The long-sought bolting gene B in the sugar beet crop has been identified. Early flowering in sugar beet terminates root growth and limits sugar beet yields [12]. Root sucrose content is a highly heritable quantitative trait in sugar beet, with genes acting in an additive fashion. The genetic investigation conducted by [13] showed that three or four loci were involved in the expression of sucrose content and [14] localized five QTLs for root sugar content on five of the nine beet chromosomes. In contrast, root weight is affected by nonadditive gene action and highly influenced by the environment, which indicates that it is difficult to predict sucrose content during the breeding process [13].

The objective of this paper was to determine genetic inheritance parameters of major quantitative properties, the mechanism of gene action controlling the inheritance of yield components, and proportional contribution of parental genotypes and their interactions to the expression of yield components. This was done in order to improve hybridization planning and exploitation of heterosis effect in diploid sugar beet hybrids.

## 2. Materials and Methods

The survey included two diploid male-sterile monogerm lines and three single (SC) male-sterile hybrids as maternal components, while three multigerm diploids were used as pollinators. Monogerm male-sterile material (2n mm MS) differed in origin and productivity properties. Apart from the parental components, 15 diploid monogerm hybrid combinations (5 × 3) were also included in the study. Field experiments with the parents and experimental hybrids were carried out in 2008 and 2009, in Aleksinac and Pancevo.

In order to determine the mode of inheritance of traits, the mean values of the hybrids were compared to those of the parents. The mean hybrid value equal to the parental average was marked as an intermediate mode of inheritance (*i*). The mean value of the hybrids closer to one of the parents was marked as partial dominance (pd). The mean value at the level of one of the parents was marked as full dominance (*d*). The significantly higher value from parents with a higher mean value or the lower value from parents with a lower mean value was interpreted as positive or negative heterosis, respectively.

The partitioning of genotypic variance into additive and dominant components was performed by half sibling (HS) and full sibling (FS) covariance.

The proportional contribution of individual components of crossbreeding (lines, testers, and interaction) to the expression of certain characteristics of F_1_ generation was calculated using the following formulas:

(1) $$\begin{array}{c} \text{Line}\;\text{contribution}\left(l\right)=\frac{\text{SS}\left(l\right)\times 100}{\;\text{SS}\left(\text{crossbreedings}\right)},\\ \text{Tester}\;\text{contribution}\left(t\right)=\frac{\text{SS}\left(t\right)\times 100}{\text{SS}\left(\text{crossbreedings}\right)},\\ \text{Interaction}\;\text{contribution}\left(lt\right)=\frac{\text{SS}\left(lt\right)\times 100}{\text{SS}\left(\text{crossbreedings}\right)}, \end{array}$$

where SS(*l*) is sum of the squares of the lines, SS(*t*) is sum of the squares of the pollinators (testers), SS(*lt*) is sum of the squares line × tester, and SS(crossbreedings) is sum of the squares of the crossbreedings.

## 3. Results and Discussion

Root yield is one of the main indicators of the value of a variety or a hybrid. It is a quantitative characteristic whose formation is determined by a greater number of genes with a weaker effect, the so-called minor genes or polygenes [15].

The highest average root yield (50.50 t ha^−1^) in maternal components was obtained by the male-sterile hybrid MS 762-1-30x1-50, while the line MS 354-17-1 had the lowest root yield (44.11 t ha^−1^). Male-sterile hybrids MS 353-5-5xP-20 and MS 762-5-15xP-5 also had high root yields (48.47 t ha^−1^ and 47.28 t ha^−1^, resp.). Pollinators (testers) differ in their genetic root yield potential, with 2340 and 2351 having the highest root yield (52.65 t ha^−1^ and 51.96 t ha^−1^, resp.) and the pollinator 2345 MR 47 having a slightly lower root yield 48.13 t ha^−1^ (Table 1).

The lines MS 354-17-1 and MS 357-9-3-3 had the highest average value of sugar content in maternal components (16.91%). Other maternal components had a lower sugar content, and the male-sterile hybrid MS 762-1-30x1-50 had the lowest value (15.43%). The pollinators had high potential for sugar content: 2345 MR 47 had the highest value of sugar content (16.91%), while 2340 and 2351 had high values as well, 16.80% and 16.70%, respectively (Table 1).

The average root yield of hybrids ranged from 52.91 t ha^−1^ for the hybrid combination MS 354-17-1x2340 to 59.65 t ha^−1^ for the hybrid (MS 353-5-5xP-20)x2345MR 47. The average root yield for both standards was 55.60 t ha^−1^ (Table 2).

All hybrid combinations, regardless of the use of pollinators, had a higher root yield compared to the better parent; that is, heterosis was manifested to a greater or lesser degree. Only one combination exhibited full dominance of parents with a higher mean value. The study conducted by [16] evaluated the effect of heterosis when crossbreeding MS lines with multigerm pollinators. They confirmed the occurrence of heterosis for root yield but not for sugar content.

Significant and highly significant differences in root yield were found by the LSD test of significance for research years, sites, and average values.

The partitioning of genotypic variance into additive and dominant components showed that the variance due to the dominant action of genes was much higher than additive variance. This means that root yield, when taking into account both parents and hybrids, is governed by the dominant action of genes with a smaller proportion of genes with an additive effect (Table 3). The findings of [17] confirmed the importance of nonadditive gene action for the inheritance of root and sugar yield. The same authors stated that the additive effect of genes is the most responsible for the inheritance of sugar content.

The average line contribution to root yield for both years was the most significant. Thus, the proportional line contribution was 55.12% in 2008 and 42.63% in 2009. The contribution of pollinators was significantly lower in 2008, while it was 24.76% in 2009. The impact of the line × pollinator interaction on root yield was also significant—it was 41.71% in 2008 and 32.61% in 2009 (Table 4). The starting contribution values for both parents are equal, as both the maternal component and the diploid pollinator confer one gene each to the F_1_ diploid hybrid. However, maternal components used in this research are commercially used in the production of triploid hybrids, which indicates that they have good combining abilities.

Sugar content is one of the main indicators of the technological values of varieties or hybrids. Bearing in mind that sugar content is a typical quantitative property, its occurrence is governed by a number of factors, with the genetic basis of lines or hybrids having the greatest impact.

The average value of sugar content in hybrids ranged from 15.95% (hybrid combination (MS 353-5-5xP-20)x2340) to 16.95% (hybrid MS 357-9-3-3x2351). The average value of sugar content for both standards was 16.64% (Table 5).

Significant and highly significant differences in sugar content were found by the LSD test of significance for research years, sites, and average values.

Different modes of inheritance of sugar content were observed in different hybrid combinations: intermediate inheritance in 6 hybrid combinations and heterosis in 2 hybrid combinations occurring closer to the parent with a lower mean value. The occurrence of positive heterosis, closer to the parent with a higher mean value, was noted in one hybrid combination. The dominance of the parents with greater mean values was observed in three hybrids. Partial dominance of the parents with a lower mean value was found in only one case. Two hybrids had full dominance of the parents with a lower mean value. The study of [1] suggested that sugar content is inherited through intermediate inheritance to super dominance (*d*/*a* = +3) towards high content. According to [3, 5], the mode of inheritance of sugar content can be intermediate inheritance, partial dominance, or full dominance of the better parent, while heterosis is manifested in a small number of hybrid combinations.

The analysis of genotypic variance components for sugar content indicates a significantly higher proportion of the dominant component of genotypic variance in relation to the additive gene effect (Table 6). According to the results of [18], the dominant effect of genes is responsible for the inheritance of sugar content. The study conducted by [1] showed that the proportional contribution of the additive effect of genes to root yield is only 2%, while both additive and nonadditive effects have an equal impact on sugar content. However, [19] it was stated that the additive effect of genes is more important than nonadditive effect for both root yield and sugar content.

The average contribution of the maternal lines to the expression of sugar content in the test hybrids was 40.69% in 2008 and 23.25% in 2009. The contribution of diploid pollinators was 32.83% in 2008 and 35.83% in 2009. The average contribution of the line × pollinator interaction to the expression of sugar content was also significant—26.48% in 2008 and 40.92% in 2009. The average contribution for both years showed almost identical values—lines 31.97%, pollinators 34.3%, and the line × pollinator interaction 33.70% (Table 7).

## 4. Conclusion

The results obtained suggest the following.

- The occurrence of heterosis in the inheritance of root yield was noted in all hybrid combinations, except in one combination where the dominance of parents with a higher mean value was observed.
- Sugar content was usually inherited through intermediate inheritance, dominance, or heterosis occurring closer to the parent with a lower mean value. The dominance of the parents with higher mean values and positive heterosis were observed as well.
- The genotypic variance components show that the nonadditive effect of genes (dominance) has a great impact on the inheritance of root yield and sugar content, while the additive effect is less significant.
- Maternal components had the greatest proportional contribution to the expression of root yield and sugar yield, while lines, pollinators, and their interactions had an equal contribution to sugar content.

## Figures and Tables

**Table 1:** Mean values of root yield and sugar content of the parental components.

Genotype	2008		2009		*M*
	Aleksinac	Pancevo	Aleksinac	Pancevo	
	Root yield (t ha^−1^)				
MS 354-17-1	19.70	48.15	40.15	68.45	44.11
MS 357-9-3-3	20.67	53.63	45.04	64.89	46.06
MS 353-5-5xP-20	18.82	59.04	42.07	73.97	48.47
MS 762-1-30x1-50	21.55	63.03	43.55	73.85	50.50
MS 762-5-15xP-5	20.67	55.33	39.78	73.33	47.28
2n MM 2340	24.67	63.70	49.85	72.37	52.65
2n MM 2345 MR 47	21.26	54.22	46.52	70.52	48.13
2n MM 2351	23.55	63.18	50.07	71.04	51.96

	Sugar content (%)				
MS 354-17-1	16.26	16.20	17.11	18.06	16.91
MS 357-9-3-3	15.84	16.28	17.39	18.13	16.91
MS 353-5-5xP-20	14.66	15.64	16.62	16.79	15.93
MS 762-1-30x1-50	14.78	15.19	15.86	15.87	15.43
MS 762-5-15xP-5	14.64	15.35	16.29	16.44	15.68
2n MM 2340	15.96	16.43	17.64	17.19	16.80
2n MM 2345 MR 47	16.20	16.63	17.49	17.32	16.91
2n MM 2351	16.18	15.93	17.33	17.36	16.70

**Table 2:** Mean values and the mode of inheritance of root yield (t ha^−1^) in hybrids.

Hybrids	2008		2009		*M*
	Aleksinac	Pancevo	Aleksinac	Pancevo	
(1) 354-17-1x2340	21.55	65.26	50.22	74.59	52.91^d^
(2) 354-17-1x2345 MR 47	26.22	63.78	50.44	76.52	54.24^h^
(3) 354-17-1x2351	26.59	64.22	53.48	77.70	55.49^h^
(4) 357-9-3-3x2340	27.04	64.45	49.78	79.19	55.11^h^
(5) 357-9-3-3x2345 MR 47	26.29	67.18	50.07	77.33	55.22^h^
(6) 357-9-3-3x2351	28.15	64.15	50.37	71.41	53.52^h^
(7) (353-5-5xP-20)x2340	28.37	66.41	51.55	83.41	57.18^h^
(8) (353-5-5xP-20)x2345 MR 47	28.96	69.78	52.07	87.78	59.65^h^
(9) (353-5-5xP-20)x2351	30.29	67.41	52.59	79.85	57.54^h^
(10) (762-1-30x1-50)x2340	26.96	63.70	51.41	85.70	56.94^h^
(11) (762-1-30x1-50)x2345 R 47	25.41	63.63	50.07	84.45	55.89^h^
(12) (762-1-30x1-50)x2351	27.78	64.52	50.52	74.29	54.28^h^
(13) (762-5-15xP-5)x2340	28.59	68.45	50.74	83.18	57.74^h^
(14) (762-5-15xP-5)x2345 MR 47	22.74	64.52	51.26	81.93	55.11^h^
(15) (762-5-15xP-5)x2351	27.11	65.33	50.89	72.22	53.89^h^

^h^Positive heterosis; ^d^Positive dominance.

**Table 3:** Genotypic variance components for root yield.

Components	2008	2009	Average
Additive	0.356	1.332	0.844
Dominant	6.673	14.787	10.730

**Table 4:** Proportional contribution (%) of the lines, pollinators, and interactions to the expression of root yield.

Average contribution	2008	2009	Average
Lines	55.12	42.63	48.87
Pollinators	3.17	24.76	13.97
Lines × pollinators	41.71	32.61	37.16

**Table 5:** Mean values and the mode of inheritance of sugar content (%) for parents and hybrids.

Hybrids	2008		2009		*M*
	Aleksinac	Pancevo	Aleksinac	Pancevo	
(1) 354-17-1x2340	15.72	16.49	17.40	17.71	16.83^i^
(2) 354-17-1x2345 MR 47	16.14	16.25	17.68	17.10	16.79^−h^
(3) 354-17-1x2351	15.96	16.28	17.25	16.78	16.57^−d^
(4) 357-9-3-3x2340	15.88	16.14	17.19	17.43	16.61^−h^
(5) 357-9-3-3x2345 MR 47	16.54	15.98	17.45	17.29	16.81^i^
(6) 357-9-3-3x2351	16.26	16.19	17.58	17.79	16.95^d^
(7) (353-5-5xP-20)x2340	15.14	14.96	16.89	16.81	15.95^−d^
(8) (353-5-5xP-20)x2345 MR 47	15.32	15.46	16.49	16.81	16.02^−pd^
(9) (353-5-5xP-20)x2351	16.20	16.15	17.45	17.45	16.81^d^
(10) (762-1-30x1-50)x2340	14.99	15.55	16.39	17.02	15.99^i^
(11) (762-1-30x1-50)x2345 MR 47	15.42	15.72	16.57	16.46	16.04^i^
(12) (762-1-30x1-50)x2351	16.26	16.28	17.53	17.67	16.94^h^
(13) (762-5-15xP-5)x2340	15.90	15.61	16.76	16.93	16.30^i^
(14) (762-5-15xP-5)x2345 MR 47	15.74	15.65	16.48	16.87	16.19^i^
(15) (762-5-15xP-5)x2351	16.09	15.99	17.58	17.77	16.86^d^

(16) Foreign standard	15.32	16.37	17.57	17.27	16.63
(17) Domestic standard	15.98	16.05	17.35	17.22	16.65

Standard average	15.65	16.21	17.46	17.24	16.64
LSD					
0.05		0.36	Cv = 1.75%		0.18
0.01		0.47			0.23

^−h^Negative heterosis; ^h^Positive heterosis; ^i^Intermediate; ^d^Positive dominance; ^−d^negative dominance; ^−pd^Negative partial dominance.

**Table 6:** Genotypic variance components for sugar content.

Components	2008	2009	Average
Additive	0.036	0.020	0.028
Dominant	0.260	0.449	0.354

**Table 7:** Proportional contribution (%) of the lines, pollinators, and interactions to the expression of sugar content.

Average contribution	2008	2009	Average
Lines	40.69	23.25	31.97
Pollinators	32.83	35.83	34.33
Lines × pollinators	26.48	40.92	33.70
